# Prenatal adherence to the Mediterranean diet decreases the risk of having a small-for-gestational-age baby, ECLIPSES study

**DOI:** 10.1038/s41598-022-17957-8

**Published:** 2022-08-13

**Authors:** Andrés Díaz-López, Sandra Díaz-Torres, Francisco Martín-Luján, Josep Basora, Victoria Arija

**Affiliations:** 1 Department of Basic Medical Sciences, Nutrition and Mental Health Research Group (NUTRISAM), Faculty of Medicine and Health Sciences, Universitat Rovira i Virgili (URV), C/Sant Llorenç 21, Reus, Spain; 2 Institut d’Investigació Sanitària Pere Virgili (IISPV), Tarragona, Spain; 3 Institut d’Investigació en Atenció Primària IDIAP Jordi Gol, Institut Català de La Salut (ICS), Barcelona, Spain; 4 Collaborative Group on Lifestyles, Nutrition and Tobacco (CENIT), Institut d’Investigació en Atenció Primària IDIAP Jordi Gol, Institut Català de la Salut (ICS), Tarragona, Spain

**Keywords:** Health care, Medical research, Risk factors

## Abstract

There is little evidence regarding the role that consuming a Mediterranean diet (MedDiet) during pregnancy plays in foetal growth. We therefore examined the relationship between maternal MedDiet adherence during pregnancy and anthropometric measures and small-for-gestational-age (SGA) at birth in a Spanish population on the north-eastern Mediterranean coast of Spain. Prospective analysis involved 614 mother–newborn pairs from the ECLIPSES study. Diet during pregnancy was assessed using a validated food frequency questionnaire, and a relative MedDiet score (rMedDiet) was calculated. Neonatal information, including weight, length, head circumference and SGA (< 10th percentile) at birth, was recorded. Multivariable logistic regression analyses were performed. The mean rMedDiet score was 9.8 (SD 2.1), ranging from 5 to 16 points. In the sample, 45% of the women had low (≤ 9 points), 32% had medium (10–11 points), and 22% had high (≥ 12 points) adherence to the rMedDiet. There was no association between rMedDiet and birth weight, length, head circumference or anthropometric indices (weight/length ratio and ponderal index). Pregnant women with a high rMedDiet score had a lower risk of delivering a SGA baby for weight (high *vs* low, OR = 0.36; 95% CI 0.16–0.79) and head circumference (high *vs* low, OR = 0.39; 95% CI 0.18–0.86), and a nonsignificant decrease in risk of SGA for length (high *vs* low, OR = 0.57; 95% CI 0.28–1.17). In conclusion, closer adherence to the MedDiet during pregnancy may have beneficial effects on foetal growth.

## Introduction

Suboptimal foetal growth has been associated with increased short- and long-term risk of metabolic and chronic diseases (e.g., obesity, insulin resistance, type 2 diabetes, and cardiovascular disease (CVD)^1–3^) as well as mortality from CVD later in life^4^. Identifying modifiable determinants of foetal growth is therefore of considerable significance for public health.

In addition to genetic factors^5^ and uteroplacental function^6^, maternal diet during pregnancy appears to be a potentially modifiable risk factor that influences the development environment of the foetus; consequently, the deficient supply of nutrients can affect foetal growth^7,8^. Pregnant women need to consume enough energy and nutrients to meet the increased nutritional requirements and to support foetal growth^9^. Notwithstanding this, compared with national recommendations, macro- and micronutrient intakes during pregnancy, in particular of docosahexaenoic acid (DHA), iron, iodine, calcium, folic acid, and vitamin D, are generally less than optimal^10,11^. It is well-documented that deficiency in pregnancy of these nutrients may impair foetal development^12^. Thus, improving the overall quality of the maternal diet and ensuring adequate nutritional status during this period is of utmost importance for optimal intra-uterine foetal growth, which in turn improves infant birth weight and length^13,14^.

There is substantial evidence regarding the effects of single nutrients, combinations of certain nutrients^8^, and/or foods^15,16^ on newborn growth parameters. However, obtaining an overall picture of the effect of maternal diet quality on birth outcomes requires dietary pattern analysis, which is an alternative and more comprehensive approach^16–18^ that examines the correlations between dietary components and other lifestyle-related habits^19^.

Consequently, there has been recent interest in the association between maternal dietary patterns during pregnancy and foetal growth. However, no clear relationship has yet been established. Some individual studies have shown that maternal intake patterns that include a high consumption of healthy regional-specific foods appear to be associated with a lower risk of low birth weight (LBW) and small-for-gestational-age (SGA) births; however, a recent literature review^16^ and a meta-analysis^17^ that analysed previous studies, including studies that focus on the Mediterranean-style diet (MedDiet)^20–27^, did not confirm these associations. Certain differences regarding cultural, social and environmental aspects are the probable reasons behind the diverse study results. Another explanation may be related to the various foetal growth measurements taken in the different studies. Nevertheless, the term SGA seems to be the best indicator of suboptimal foetal growth, since the sex and gestational age of the infant are taken into account^28,29^, in addition to being the most widely used as the outcome in previous studies^16^. Moreover, foetal growth may depend on maternal factors other than prenatal dietary pattern, such as age, pre-pregnancy BMI, gestational weight gain (GWG), educational level, social class, and smoking. Previous studies have rarely reported subgroup analyses, which increases the possibility of residual confounding data. Further research is therefore needed to better understand the association, if any, between the maternal MedDiet pattern and foetal growth. Therefore, in this study we prospectively examined the relationship between maternal adherence to the MedDiet during pregnancy and anthropometric measurements or indices of the newborn (weight, length, head circumference (HC), weight-length ratio and ponderal index) and SGA at birth, in a large Spanish mother–child cohort on the north-eastern Mediterranean coast of Spain.

## Methods

### Study design and participants

Longitudinal population-based study analysing data from healthy pregnant women who participated in the ECLIPSES trial as well as data from their children. Details of the study's protocol have been described elsewhere^30^. Briefly, the ECLIPSES is an ongoing mother–child cohort study that evaluates the long-term impact of dietary, psychological and environmental factors during pregnancy on offspring outcomes (including physical and neurobehavioral development)^30^. A total of 793 pregnant women were recruited during the first prenatal visit (before the 12th gestational week (GW)) by midwives from 12 sexual and reproductive health care services (ASSIR) of the Catalan Institute of Health (ICS) in the province of Tarragona (Catalonia, Spain), between 2013 and 2017. Eligible participants were healthy adult women older than 18 years with ≤ 12 weeks of gestation. Further details of the inclusion/exclusion criteria can be found elsewhere^30^. The ECLIPSES trial is registered on both the ClinicalTrials.gov (identification number NCT03196882) and the EU Clinical Trials Register (EUCTR-2012-005480-28). This study was approved by the Ethical Committee of the Jordi Gol Institute for Primary Care Research (IDIAP) and the Pere Virgili Institute for Health Research (IISPV). All participants signed an informed consent form. The study complies with the tenets of the Declaration of Helsinki.

### Dietary assessment

Eating habits were assessed through a self-administered food frequency questionnaire (FFQ) regarding 45-food groups, previously validated in our population^31^. Women reported usual food consumption in each trimester of pregnancy (at weeks 12, 24, and 36 gestation). The FFQs were explained and collected by specialized midwives. Nutritionists then reviewed the questionnaires and recorded the food data. Women reported their usual frequency of consumption of foods and drinks per week or per month. The average consumption rations of each item were obtained and compared with the dietary guidelines of the Sociedad Española de Nutrición Comunitaria (SENC)^32^. Each food item was then converted into consumption in grams per day by applying the average consumption ration for our population according to data previously obtained in the consumption surveys conducted by the research group^31^; for example: milk (220 g), salad (100 g), legumes (60 g), eggs (55 g), meat (150 g), fish (150 g), fruit (100 g), among others. From this information we estimated total daily energy intake and macro- and micronutrients by primarily using the REGAL food composition table^33^ and other tables published for Spanish foods^34^.

We were interested in the effects of adherence to the MedDiet during pregnancy, which we assessed by using the relative MedDiet (rMedDiet) score based on the intake of nine components: fruits (including nuts and seeds but excluding fruit juices), vegetables, legumes, cereals (including whole-grain and refined flour, pasta, rice, other grains), fresh fish (including prawns and shellfish), meat (including processed meat), dairy products (including milk, yogurt, cheese, and cream desserts), olive oil, and alcohol. This score is a modified version of the original Mediterranean Diet Score^35^, which has been previously used in a pregnant population^36^. All the food groups (except alcohol) were measured as g/1000 kcal/day, and were divided into tertiles. The lowest tertile was coded as 0, the medium tertile as 1 and the highest as 2 for fruits, vegetables, legumes, cereals, fresh fish and olive oil. Conversely, the lowest tertile was coded as 2, medium as 1 and highest as 0 for meat and dairy products. Alcohol intake, considered as harmful during pregnancy, was scored as a dichotomous variable and coded with 0 for women who consumed alcohol and 2 for women who did not drink alcohol. After adding together the scores for each component, the resulting score ranged from 0 to 18 points, with higher scores indicating a greater adherence to MedDiet, and therefore, higher diet quality. For the purpose of these analyses, we assessed the adherence to MedDiet throughout pregnancy (n = 614 in the first; n = 493 in the second and n = 440 in the third trimesters). If dietary data was not available in the second and third trimesters of pregnancy, the average score was calculated using the available data. Because there are no pre-established cut-off points for the pregnant population, the score was divided into tertiles to reflect low (tertile 1, 0–9 points), medium (tertile 2, 10–11 points), and high (tertile 3, 12–18 points) adherence to the rMedDiet.

### Newborn anthropometric measurements

Infant’s sex (male, female) and anthropometric measurements, including body weight (g), height (cm), and HC (cm) were measured at birth. Neonatal anthropometric parameters were measured just after birth by the obstetrician or midwife using standard procedures. These measurements were used to calculate the weight to length ratio (g/cm) and Ponderal Index (PI) using the established equation PI = weight (g) × 100)/length^3^ (cm). Gestational age at delivery was established by obstetricians based on the first day of the last menstrual period reported at recruitment and corrected by ultrasound measurements recorded at about 12 GW.

Outcome variables used in the current study were birth weight, birth length, birth HC, anthropometric indices (weight-length ratio and PI), and SGA at birth, the criteria for which were newborns with birth anthropometric measurements below the 10th percentile according to the gestational age- and sex-specific reference growth curves based on INTERGROWTH-21st international standards^37^.

### Assessment of covariates

We recorded information regarding maternal age, socio-economic level, educational level (primary, secondary and university studies), lifestyle habits (including physical activity, smoking and alcohol consumption), and medical and obstetric history (including parity, planned pregnancy, and type of delivery (at birth)) in the first trimester of pregnancy (at week 12). Midwives and nutritionists compiled this information during personal interviews and from specific questionnaires. Socioeconomic level was calculated according to occupational status using the Catalan classification of occupations (CCO-2011)^38^ and was classified as low, middle, high. Physical activity was measured using the short version of the International Physical Activity Questionnaire (IPAQ-S)^39^. At enrolment and each trimester thereafter, maternal weight (in kg to the nearest 0.1 kg) and height (in cm to the nearest 0.1 cm) were also measured. Based on the criteria proposed by the WHO^40^, the body mass index (BMI) was calculated as weight/height squared (kg/m^2^) and women were classified as normal weight (BMI < 25 kg/m^2^) or excess weight (BMI ≥ 25 kg/m^2^). Total GWG was calculated and conditioned by the initial BMI was categorized into insufficient, adequate or excessive GWG according to the 2009 IOM recommendations^41^.

### Statistical analysis

Descriptive statistics were expressed as mean ± SD for quantitative variables and number of women (%) for categorical variables. ANOVA and the chi-square test were used, as appropriate, to test differences in baseline characteristics across categories of rMedDiet adherence score (low, medium, and high adherence).

The associations between the maternal rMeDiet adherence score and SGA for birth weight, length and HC were assessed using logistic regression models with rMeDiet as both a categorical (lowest category as reference) and a continuous variable. In the latter case, we calculated the associations of SGA risk with a 1-point increase in the rMeDiet score. Estimates were presented as odds ratios (OR) and 95% confidence intervals (CIs). We fitted a crude univariate model and, based on existing literature, a multivariable model for each outcome variable with adjustment for following potential outcome and exposure-related confounders: maternal age (years, continuous) and total energy intake (kcal/day, continuous); pre-pregnancy BMI (kg/m^2^, continuous), GWG (insufficient, adequate, excessive) educational level (primary or lower, secondary, university) smoking during pregnancy (yes, no), social class (low, middle, high), type of delivery (normal vaginal, caesarean), primiparous (yes, no), and planned pregnancy (yes, no). Tests for linear trends across categories were conducted by modelling the median value for the rMeDiet adherence score categories as continuous variables.

The potential effect modification was assessed through stratified analyses for maternal age (< 30, ≥ 30 years), pre-pregnancy BMI (normal weight, excess weight), GWG (insufficient, adequate, excessive), educational levels (primary or lower/secondary, university), social class (low/middle, high), smoking during pregnancy (yes, no), and having a planned pregnancy (yes, no). Multivariable-adjusted OR (95% CIs) per one-point increase in maternal rMeDiet score was presented. Interactions were tested with the likelihood ratio tests, which involved comparing models with and without cross-product terms, and were not significant at the significance level of ≤ 0.05.

Finally, as a sensitivity analysis to assess the consistency of our results, we repeated the main analysis but this time excluding premature newborns (gestational age < 37 weeks, n = 19 infants, 1.6% of total).

All statistical analyses were carried out using the 15.0 version of the statistical software STATA (StataCorp LP, Texas, USA). Statistical significance was set at p < 0.05.

### Ethical approval

All procedures performed in the study were in accordance with the ethical standards of the Institut d'Investigació en Atenció Primaria de Salut (IDIAP) and the Institut d'Investigació Sanitària Pere Virgili (IISPV) and with the 1964 Helsinki declaration and its later amendments or comparable ethical standards.

## Results

The study sample consisted of 614 mothers and their babies (51% girls), from whom data was taken regarding the main variables of interest, these being maternal diet (at least in the first trimester of pregnancy) and the infant’s anthropometric measurements at birth. Table 1 shows the characteristics of the mothers and newborns according to the maternal rMeDiet adherence score categories during pregnancy. Overall, the mean age of the mothers was 30.5 (SD 5.1) years and pre-pregnancy BMI was 25.1 (SD 4.5) kg/m^2^. Approximately 30% of mothers had a university education, 19% were of high social class, and 17% of them smoked during pregnancy. There were no significant differences in these characteristics between women included in the analysis versus those who were excluded (all p < 0.05, data not shown).

**Table 1:** General maternal and child characteristics according to Mediterranean Diet adherence categories during pregnancy (n = 614).

	Total cohort	rMedDiet adherence categories			*p* value
		Low (0–9 points)	Medium (10–11 points)	High (12–18 points)	
**Maternal characteristics**					
n (%)	614 (100)	279 (45)	199 (33)	136 (22)	
Age (years), mean (SD)	30.5 (5.1)	29.5 (5.2)	31.0 (5.0)	31.9 (4.8)	< 0.001
Age categories (years), n (%)					
< 25	82 (13)	50 (18)	24 (12)	8 (8)	
25–29	163 (27)	85 (30)	45 (23)	33 (24)	0.001
≥ 30	369 (60)	144 (52)	130 (65)	95 (70)	
Weight (kg), mean (SD)	65.9 (11.8)	65.9 (12.1)	65.7 (11.6)	66.0 (11.6)	0.97
Pre-pregnancy BMI (kg/m^2^), mean (SD)	25.1 (4.5)	25.2 (4.5)	25.1 (4.5)	25.1 (4.4)	0.96
Pre-pregnancy BMI categories, n (%)					
Normal weight	354 (58)	159 (57)	117 (59)	78 (57)	0.92
Excess weight	260 (42)	120 (43)	82 (41)	58 (43)	
Gestational weight gain (kg), mean (SD)	10.4 (3.8)	10.5 (3.8)	10.1 (4.1)	10.5 (3.4)	0.45
IOM GWG recommendations, n (%)*					
Insufficient	252 (41)	105 (37)	96 (48)	51 (38)	0.035
Adequate	240 (39)	124 (44)	65 (33)	51 (38)	
Excessive	122 (20)	50 (18)	38 (19)	34 (25)	
Physical activity (MET/min/w), mean (SD)	686.7 (925.1)	716.8 (979.8)	611.1 (825.1)	735.5 (945.9)	0.36
Educational level, n (%)					
Primary or less	196 (32)	93 (33)	66 (33)	37 (27)	
Secondary	231 (38)	121 (44)	69 (35)	41 (30)	0.001
University	187 (30)	65 (23)	64 (32)	58 (43)	
Social class, n (%)					
Low	112 (18)	55 (20)	43 (22)	14 (10)	
Middle	383 (63)	187 (67)	108 (54)	88 (65)	0.001
High	119 (19)	37 (13)	48 (24)	34 (25)	
Smoking, n (%)					
No	509 (83)	224 (80)	173 (87)	112 (82)	0.16
Yes	105 (17)	55 (20)	26 (13)	24 (18)	
Type of delivery, n (%)					
Normal vaginal	528 (86)	244 (87)	171 (86)	113 (83)	0.48
Caesarean	86 (14)	35 (13)	28 (15)	23 (17)	
Parity, n (%)					
Primiparous	363 (59)	162 (58)	126 (63)	75 (55)	0.29
Multiparous	251 (41)	117 (42)	73 (37)	61 (44)	
Planned pregnancy, n (%)					
Yes	490 (80)	217 (78)	158 (80)	115 (85)	0.21
No	122 (20)	62 (22)	40 (20)	20 (15)	
**Newborn characteristics**					
Infant’s sex, n (%)					
Female	311 (51)	129 (46)	113 (57)	69 (51)	0.08
Male	303 (49)	151 (53)	86 (43)	67 (49)	
Birth weight (g), mean (SD)	3294.6 (460.1)	3270.7 (447.4)	3297.1 (473.5)	3338.9 (407.1)	0.36
Birth length (cm), mean (SD)^†^	49.3 (2.1)	49.3 (2.1)	49.2 (2.1)	49.6 (1.9)	0.30
Birth HC (cm), mean (SD)^‡^	34.5 (1.5)	34.4 (1.5)	34.5 (1.3)	34.7 (1.7)	0.29
Weight to length ratio (g/cm)^†^	66.9 (7.3)	66.8 (7.7)	67.0 (7.1)	67.1 (6.8)	0.94
Ponderal index (g/cm^3^)^†^	2.75 (0.29)	2.75 (0.27)	2.77 (0.32)	2.73 (0.27)	0.49
GA at delivery (weeks), mean (SD)	39.6 (1.5)	39.7 (1.5)	39.6 (1.5)	39.7 (1.4)	0.54

Values are expressed as a mean (± SD, standar desviation) or number (%).

*BMI* body mass index, *GWG* gestational weight gain, *rMedDiet* Mediterranean diet, *HC* head circumference, *GA* gestational age.

p values for differences across the three MedDiet adherence categories were calculated with the Chi-square test or ANOVA.

*Recommendations for GWG by the IOM guidelines are for initial BMI < 18.5 kg/m^2^ total weight gain between 12.5–18 kg; for BMI of 18.5 to 24.9 kg/m^2^ total weight gain between 11.5 and 16 kg; for BMI of 25.0–29.9 kg/m^2^ total weight gain between 7 and 11.5 kg; and for BMI ≥ 30 kg/m^2^ total weight gain between 5 and 9 kg.

^†^n = 495.

^‡^n = 368.

The rMedDiet scores ranged from 5 to 16 points with the mean score being 9.8 (SD 2.1). Out of 614 women, only 22% reported high adherence (≥ 12 points) to the rMedDiet. Women with a high adherence to the rMedDiet during pregnancy were more likely to be older and gain excess gestational weight, and had a higher level of education and social class (Table 1).

Overall, the mean gestation was 39.6 (SD 1.4) weeks. The mean birth weight was 3294.6 (SD 460.1) g, mean length was 49.3 (SD 2.1) cm, and mean HC was 34.5 (SD 1.5) cm in the population. All neonatal anthropometric measures were significantly higher (p < 0.005) in boys (3370.1 (SD 442.4) g, 49.6 (SD 2.2) cm, 34.8 (SD 1.7) cm) than in girls (3221.1 (SD 465.8) g, 49.0 (SD 1.9) cm, 34.3 (SD 1.3) cm). As shown in Table 1, no statistically significant differences between maternal rMedDiet adherence categories were found regarding newborn anthropometric measurements (birth weight, length, and HC) or indices (weight to length ratio and PI). Likewise, the findings remained unchanged in all of the anthropometric parameters when we carried out separate multivariate linear regression analyses adjusted for all confounders listed above plus the infant’s sex and gestational age, with the lowest rMedDiet category used as a reference (data not shown).

As shown in Table 2, the distribution of food groups and nutrient intakes by tertiles of the maternal rMedDiet score was generally in the expected direction. Women with high adherence to the rMedDiet showed significantly higher intake of food items typical of this pattern and a more favourable overall nutritional profile.

**Table 2:** Maternal consumption of food groups, and energy and nutrient intake according to Mediterranean Diet adherence categories during pregnancy (n = 614).

	Total cohort	rMedDiet adherence categories			*p* value
		Low (0–9)	Medium (10–11)	High (12–18)	
rMedDiet score (point)*	10.0 (3.0)	8.0 (2.0)	10.0 (1.0)	13.0 (1.0)	
rMedDiet component score (g/day)*					
Fruits and nuts	165.1 (127.9)	136.9 (109.7)	170.9 (123.2)^‡^	211.5 (147.7)^‡^	< 0.001
Vegetables	74.2 (48.1)	58.1 (37.6)	78.2 (40.0)^‡^	99.8 (48.3)^‡^	< 0.001
Legumes	13.5 (10.0)	11.4 (8.6)	14.1 (11.4)^‡^	17.1 (12.8)^‡^	< 0.001
Cereals	150.2 (65.9)	141.8 (59.1)	155.1 (67.9)	157.3 (73.6)^‡^	0.015
Fresh fish and shellfish	42.8 (32.5)	33.8 (26.8)	45.2 (31.54)^‡^	58.8 (33.5)^‡^	< 0.001
Meat and meat products	99.1 (46.7)	107.1 (50.0)	96.4 (41.6)^‡^	91.7 (40.4)^‡^	< 0.001
Dairy products	328.9 (148.4)	351.3 (127.7)	322.6 (161.9)	266.7 (180.7)^‡^	< 0.001
Alcohol	0.0 (0.0)	0.0 (0.0)	0.0 (0.0)	0.0 (0.0)^‡^	0.002
Olive oil	69.4 (23.5)	71.8 (23.3)	67.5 (24.3)	67.0 (23.5)	0.41
**Energy and dietary nutrient intakes/day**					
Energy (kcal)	2116.5 (626.0)	2157.2 (555.2)	2099.0 (659.7)	2019.0 (673.4)	0.07
Proteins (g)	55.9 (18.2)	55.7 (17.9)	56.2 (17.7)	56.3 (22.1)	0.64
Carbohydrates (g)	168.3 (69.2)	170.4 (64.9)	170.7 (60.9)	161.2 (79.9)	0.82
Total lipids (g)	103.7 (20.3)	106.6 (19.5)	101.8 (19.5)^‡^	99.6 (21.3)^‡^	0.016
Saturated fats (g)	26.7 (6.9)	27.8 (6.8)	25.9 (70.1)^‡^	25.1 (6.6)^‡^	< 0.001
Monounsaturated fats (g)	58.1 (10.7)	60.3 (9.5)	56.7 (9.5)^‡^	56.2 (11.8)^‡^	0.012
Polyunsaturated fats (g)	11.0 (1.6)	10.9 (1.5)	10.9 (1.5)	11.3 (1.8)^‡^	0.038
Fibre (g)	12.0 (4.6)	11.2 (4.1)	12.4 (4.7)^‡^	13.6 (5.2)^‡^	< 0.001
Calcium (mg/d)	668.4 (251.1)	672.5 (225.6)	672.1 (255.1)	613.7 (274.9)^‡^	0.009
Iron (mg)	7.4 (2.5)	7.3 (2.2)	7.5 (2.5)	7.6 (2.9)^‡^	0.020
Vitamin A (µg)	610.0 (198.8)	593.3 (197.2)	613.1 (194.2)	613.5 (244.3)^‡^	0.018
Vitamin E (mg)	10.8 (1.9)	10.8 (1.7)	10.8 (1.9)	11.2 (2.6)^‡^	< 0.001
Vitamin C (mg)	70.1 (38.7)	63.5 (30.4)	74.8 (37.2)^‡^	89.1 (45.9)^‡^	< 0.001
Beta-carotene (μg)	1573.1 (584.7)	1334.1 (464.5)	1639.9 (536.2)^‡^	1972.8 (635.0)^‡^	< 0.001
Vitamin D (µg)	1.7 (1.1)	1.5 (1.0)	1.8 (1.1)^‡^	2.1 (1.0)^‡^	< 0.001
Vitamin B6 (mg)	1.2 (0.4)	1.2 (0.4)	1.2 (0.4)	1.3 (0.5)^‡^	0.001
Vitamin B12 (µg)	4.3 (1.7)	4.3 (1.7)	4.3 (1.5)	4.2 (1.9)	0.69
Folate (μg)	193.4 (72.2)	181.8 (59.4)	199.4 (72.8)^‡^	219.5 (98.1)^‡^	< 0.001

*Values are expressed in median values (interquartile range). rMedDiet, Mediterranean diet.

^‡^p value < 0.05 vs. low category. p values for differences across the three rMedDiet adherence categories were calculated with ANOVA.

Overall, the prevalence of SGA for birth weight, length and HC was 10.7% (12.8% males, 8.6% females), 13.3% (17.3% males, 9.3% females) and 16.8% (16.8% males, 16.7% females), respectively.

In the unadjusted model, compared to women in the lowest tertile of rMeDiet adherence during pregnancy, those in the top tertile showed a significantly lower risk of delivering a SGA baby in terms of weight (high-rMeDiet vs. low-rMeDiet, OR = 0.40; 95% CI 0.18–0.85; p = 0.017) and in terms of HC (high-rMeDiet vs. low-rMeDiet, OR = 0.44; 95% CI 0.21–0.91; p = 0.028), and a nonsignificant decrease in risk of SGA in terms of length (high- rMeDiet vs. low-rMeDiet, OR = 0.66, 95% CI 0.33–1.29; p = 0.225). The associations were strengthened for risk of SGA in terms of birth weight (multivariable-adjusted model, high- rMeDiet vs. low-rMeDiet, OR = 0.36; 95% CI 0.16–0.79) and SGA in terms of HC (multivariable-adjusted model, high- rMeDiet vs. low-rMeDiet, OR = 0.39, 95% CI 0.18–0.86) after adjustment for potential confounders (Table 3). The same pattern was observed when the rMedDiet score was modelled as a continuous variable for each one-point increment (multivariable-adjusted model, OR = 0.74; 95% CI 0.64–0.85 and OR = 0.82; 95% CI 0.72–0.94 for SGA for weight and HC, respectively). We also re-ran the analyses after excluding premature newborns but found no change in the results (data not shown). Moreover, the maternal rMedDiet score was not associated with SGA in terms of length when analysed by category or in a continuous way, in any of the models (Table 3).

**Table 3:** Multivariable-adjusted Odds ratios (ORs) and 95% confidence intervals (95% CIs) for associations between Mediterranean diet categories during pregnancy and SAG (< P_10_) in weight, length and head circumference (n = 614).

	SGA (< P_10_) for weight		SGA (< P_10_) for length^†^		SGA (< P_10_) for HC^‡^	
	N (%)	Adjusted OR (95% CI)	N (%)	Adjusted OR (95% CI)	N (%)	Adjusted OR (95% CI)
**rMedDiet adherence score (point)**						
Continuous (per 1-point increase)		0.74 (0.64–0.85)*		0.91 (0.78–1.03)		0.82 (0.72–0.94)*
Low (0–9)	42 (15)	1 (ref.)	35 (16)	1 (ref.)	37 (22)	1 (ref.)
Medium (10–11)	15 (8)	0.42 (0.22–0.81)*	18 (11)	0.65 (0.34–1.22)	17 (14)	0.56 (0.29–1.08)
High (12–18)	9 (7)	0.36 (0.16–0.79)*	13 (11)	0.57 (0.28–1.17)	11 (11)	0.39 (0.18–0.86)*
*P*-trend		0.005		< 0.013		0.015
**Age categories (years)**						
< 25	9 (11)	0.94 (0.37–2.35)	6 (10)	1.20 (0.42–3.45)	11 (25)	1.20 (0.42–3.45)
25–29	15 (9)	1 (ref.)	13 (11)	1 (ref.)	16 (18)	1 (ref.)
≥ 30	42 (11)	1.48 (0.62–3.51)	47 (15)	2.19 (0.82–5.87)	38 (15)	0.71 (0.28–1.79)
Pre-pregnancy BMI (kg/m^2^)	66 (11)	1.00 (0.94–1.07)	66 (13)	0.96 (0.89–1.03)	65 (17)	1.02 (0.95–1.08)
**IOM GWG recommendations**						
Insufficient	34 (14)	2.16 (1.14–4.09)*	30 (14)	1.12 (0.61–2.06)	27 (16)	0.96 (0.50–1.81)
Adequate	18 (8)	1 (ref.)	24 (13)	1 (ref.)	25 (17)	1 (ref.)
Excessive	14 (11)	1.77 (0.81–3.87)	12 (13)	1.20 (0.55–2.64)	13 (18)	1.02 (0.46–2.25)
**Physical activity (MET/min/week)**						
Educational level						
Primary or less	22 (11)	1 (ref.)	23 (16)	1 (ref.)	22 (20)	1 (ref.)
Secondary	27 (12)	0.87 (0.45–1.68)	21 (12)	0.58 (0.29–1.15)	22 (16)	0.70 (0.34–1.43)
University	17 (9)	0.70 (0.29–1.69)	22 (13)	0.67 (0.30–1.51)	21 (15)	0.89 (0.38–2.11)
Social class						
Low	12 (11)	1 (ref.)	8 (10)	1 (ref.)	11 (22)	1 (ref.)
Middle	41 (11)	0.99 (0.47–2.08)	46 (15)	1.62 (0.69–3.82)	42 (16)	0.84 (0.36–1.93)
High	13 (11)	1.27 (0.44–3.63)	12 (12)	1.26 (0.40–4.01)	12 (16)	1.42 (0.43–4.66)
Smoking						
No	44 (9)	1 (ref.)	52 (12)	1 (ref.)	45 (14)	1 (ref.)
Yes	22 (21)	2.62 (1.44–4.78)*	14 (18)	1.41 (0.71–2.81)	20 (33)	3.64 (1.83–7.23)*
Parity						
Primiparous	34 (9)	1 (ref.)	28 (13)	1 (ref.)	30 (18)	1 (ref.)
Multiparous	32 (13)	2.62 (0.94–2.96)	38 (13)	1.16 (0.66–2.05)	35 (16)	0.97 (0.52–1.76)
Planned pregnancy						
No	15 (12)	1 (ref.)	28 (13)	1 (ref.)	16 (21)	1 (ref.)
Yes	51 (10)	0.89 (0.46–1.72)	38 (13)	0.76 (0.40–1.45)	49 (16)	0.89 (0.45–1.74)
Energy intake (Kcal/day)	66 (11)	0.99 (0.99–1.00)	66 (13)	1.00 (0.99–1.00)	65 (17)	1.00 (0.99–1.00)

Logistic regression models were used to calculate Odds ratios (OR) and 95% confidence intervals (IC al 95%).

BMI, body mass index; SGA, small-for-gestational-age; rMedDiet, Mediterranean diet; P_10,_ percentile 10. Logistic regression models were mutually adjusted for all characteristics displayed in this table.

*p value < 0.05.

^†^n = 495.

^‡^n = 368.

**Figure 1:**
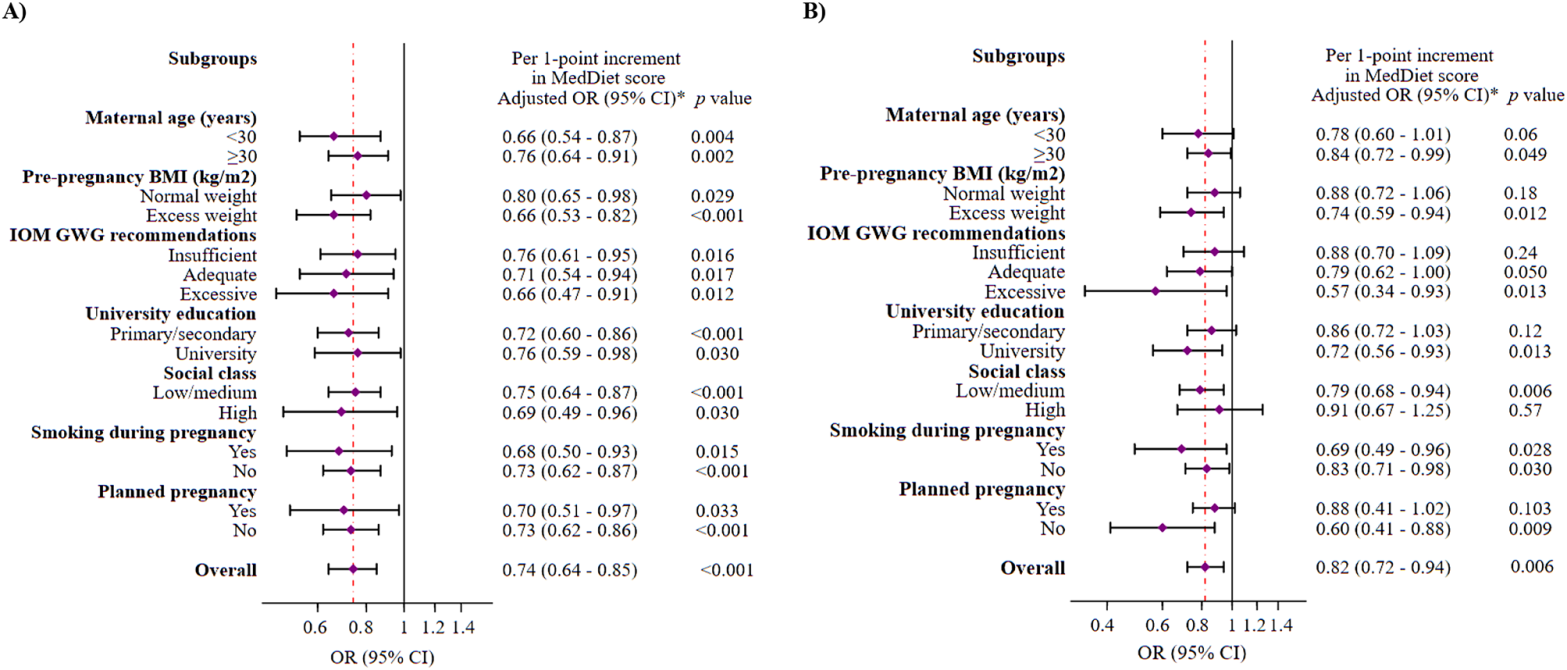
Multivariable-adjusted ORs (95% CIs) of SAG (< P_10_) infant at birth in weight (**A**) and head circumference (**B**) associated with a 1-point increase in the maternal rMedDiet score for selected subgroups. *Models were adjusted for age (< 25, 25–29, ≥ 30), energy intake (Kcal/day), BMI (kg/m^2^), IOM GWG recommendations (insufficient, adequate, excessive), educational level (primary or lower, secondary, university) smoking (yes, no), social class (low, middle, high), type of delivery (normal vaginal, caesarean) primiparous (yes, no), and planned pregnancy (yes, no), except for the variables used as subgroup variables in each case. The diamonds represent OR and the whisker plots represent 95% CIs. *BMI* body mass index, *GWG* gestational weight gain, *rMedDiet* Mediterranean diet.

Figure 1 shows that stratified analyses of different variables of interest further confirm the associations observed between the rMeDiet score and the risk of SGA in terms of weight and HC in the original analysis.

## Discussion

In the present study, we found that pregnant women with a high rMedDiet adherence score had a significantly lower risk of having a SGA (< 10th percentile) infant in terms of weight and HC, and a nonsignificant decrease in risk of SGA in terms of length. Our study adds to current knowledge regarding the influence of maternal diet on foetal growth and highlights the importance of promoting a healthy MedDiet eating pattern early in pregnancy as an effective strategy for preventing SGA births.

Nevertheless, the findings of various studies regarding specific dietary patterns during pregnancy and their effects on newborn growth parameters have been heterogeneous and inconclusive, including those that have analysed the Mediterranean-style diet^16,17^. Importantly, dietary patterns and the content of the specific food groups consumed are subject to regional variations that are influenced by sociocultural and geographical differences. This could explain in part the lack of consensus on this subject matter.

Our results on SGA (< 10th percentile) weight, the most frequently used indicator of foetal growth restriction (FGR), are generally consistent with previous studies conducted in the Mediterranean area that have explored the possible relationship between maternal Mediterranean-style diet adherence and foetal growth^25–27^. For example, recently Martínez-Galiano et al.^25^ in a case–control study in Spain (518 SGA and 518 controls) with dietary assessment at the beginning of pregnancy, reported that women with high MedDiet adherence were less likely to deliver SGA infants, regardless of the index used to evaluate the MedDiet adherence (PREDIMED, Trichopoulou and Panagiotakos). Similarly, in two large mother–child cohorts in Spain (INMA-Atlantic and INMA-Mediterranean, n = 2461) and Greece (RHEA, n = 889)^26^, an inverse association with greater adherence to a MedDiet in early pregnancy was observed for risk of SGA in INMA-Mediterranean, but no significant associations were reported in INMA-Atlantic and RHEA cohorts. Food group intakes and the mean MedDiet score differed significantly across cohorts, which is possibly the reason for the diversity of results. Another Greek study^27^, based on a small sample (n = 82) with a FFQ applied at the end of gestation, found that poor maternal adherence to the MedDiet was associated with greater risk of intrauterine growth-restriction, and lower z-scores of birth weight and BMI.

However, the data published from areas outside the Mediterranean basin were not completely consistent. Supporting our results, a recent prospective cohort study (n = 1948) in the USA found that greater maternal adherence to a Mediterranean-style diet in early pregnancy was related to a reduced risk of SGA and LBW^42^. Also in the USA, in the Boston Birth Cohort (BBC) study (n = 8507)^21^ the MedDiet was inversely associated with risk of LBW and SGA, although the last relationship did not reach statistical significance. In another study carried out in the Generation R cohort of 3207 Caucasian pregnant mothers in Rotterdam^20^, low adherence to the MedDiet in early pregnancy was also associated with decreased intra-uterine size and lower weight at birth. However, in contrast to our results, the Infant Feeding Practices Study II^22^ of a prospective cohort of 893 pregnant US women found a nonsignificant decrease in SGA risk with greater adherence to a MedDiet during the third trimester. A study conducted in Guadeloupe (French West Indies), the TIMOUN Mother–Child Cohort study (n = 728)^23^, found no association between MedDiet adherence during pregnancy and risk of FGR in the entire sample of women. However, the authors reported a slight decrease in the risk of FGR associated with a higher MedDiet score among underweight and normal-weight women (P heterogeneity < 0.01)^23^, which is in line with our observations. Interestingly, our study found negative associations with SGA in both normal-weight and overweight pregnant women with higher adherence to the MedDiet during pregnancy. This suggests that high adherence to this pattern might modify the detrimental impact of maternal BMI on foetal growth.

It is notable that our results are also further supported by another study that focuses on maternal diet patterns which share certain similarities with the MedDiet pattern. Knudsen et al.^43^ in a large population-based mother–child cohort study of almost 45,000 Danish women found that a “health conscious” antenatal dietary pattern of vegetables, fruits, poultry and fish was associated with lower chances of SGA when compared to the “Western” pattern. Another study^44^ involving 2000 pregnant women in New Zealand showed that mothers who had higher scores in a ‘traditional’ diet rich in fruit, vegetables, and dairy products in early pregnancy were less likely to deliver a SGA infant. Finally, a recently published US study (n = 862)^24^ showed that a higher quality maternal diet, which was regarded as maternal adherence to the Alternative Healthy Eating Index during early pregnancy, was associated with decreased SGA risk.

These findings taken together with our current analysis suggest that healthy dietary patterns during pregnancy and in particular the MedDiet or combinations of higher intakes of food items typical of this pattern may be effective in preventing SGA at birth.

We have also observed that having a high adherence to the rMedDeit was also associated with a reduced risk of SGA for HC. As with infants born with a low weight, infants with a small HC at birth are also susceptible to a higher risk of non-optimal neurodevelopment, poor academic performance, low social competence and increased behavioural problems later in life^45^. Contrary to our findings, a study within the INMA and RHEA cohorts^26^ found no association between maternal MedDiet and SGA for HC. In the Generation R study, a trend towards a smaller HC at birth was observed for women with low MedDiet adherence in late pregnancy; however, associations were not significant after adjusting for confounders^20^. The limited amount of evidence in this field makes it difficult to draw definitive conclusions, and further research is required.

Our main focus in studying the rMedDiet pattern was on diet quality as a whole. In our study, high compliance with this healthy diet pattern not only consisted, as expected, of a higher consumption of fruits and nuts, vegetables, legumes, cereals, fresh fish and seafood, and a lower consumption of meat, processed meat, and dairy products, reflecting the traditional MedDiet pattern, but also resulted in a more favourable overall nutritional profile among pregnant women. Notably, these women consumed relatively more polyunsaturated fatty acid, fibre, iron, antioxidant vitamins (vitamins A and E) and beta-carotene, vitamin D, B6, and folate. All these are key nutrients that have been shown to be important for achieving appropriate foetal growth and development^8^, which explains in part the associations that we observed. For instance, inflammatory processes and oxidative stress in early pregnancy can interfere with normal placentation, which can adversely affect foetal development^46^. A maternal diet rich in vegetables and fruits in pregnancy, sources of vitamins A and E, and beta-carotene with anti-inflammatory and antioxidant properties, may protect against oxidative damage and inflammation^47^ and, subsequently, decrease the risk of SGA. In addition, it has been suggested that omega-3 fatty acids EPA and DHA have beneficial effects for increasing birthweight and reducing the incidence of SGA^48^. This effect is probably linked to their influence on placental blood flow^7^. As has been described, PUFA are also critical fatty acids for the development of most organs, but their major impact is on the foetal development of brain. Moreover, it has been shown that inadequate maternal intake of other micronutrients can also increase the risk of SGA birth. Folate, iron, and vitamin D, among others, play a crucial role as substrates and cofactors in multiple pathways of cellular processes, including synthesis of nucleic acids and cellular division, red blood cell production and enzyme activity. Their deficiency in these mechanisms can lead to foetal growth retardation that results in reduced birth size among neonates^8^. In contrast, a larger consumption of foods that do not fit the traditional MedDiet, such as red and processed meat, dairy products, refined grains, and sweetened and alcoholic beverages, might positively impact SGA risk^16,17^.

It is important to consider that infant birth size is the end-point of different growth patterns determined by multiple maternal environmental factors, regardless of genetics, which in turn enhances the possibility of residual confounding. In contrast to previous studies in this field, we carried out stratified analyses to assess how maternal age, pre-pregnancy BMI, GWG, education level, social class, smoking during pregnancy, and planned pregnancy influence the effect of the rMedDiet. We found that all subgroups showed similar trends regarding the link between greater adherence to a rMedDiet and a decreased risk of SGA weight and HC. In the present study, we also supported other environmental factors that have been considered important contributors for SGA risk, such as GWG and, in particular, insufficient maternal weight gain. The women with insufficient GWG had a more than 2.5-fold increased risk of SGA for weight compared to the group with adequate GWG within the IOM guidelines, which is consistent with a large number of previous studies^49^. Importantly, adjustment of our analyses for potential confounders linked to SGA did not change the associations obtained. Therefore, we can confirm that our results are robust under different modelling approaches.

Our study has certain strengths. First of all, the study included a relatively large sample size and a prospective design, leading to a much greater likelihood of reliable conclusions. In addition, we conducted subgroup analyses, which have seldom been performed in previous studies, and we employed a widely used rMedDiet score, thus strengthening the credibility of our observations. Nevertheless, we also acknowledge some limitations. Our study involved healthy pregnant women living in the Mediterranean region, which restricts the generalizability of our results to other study populations or non-Mediterranean settings. Another limitation of the study, as in most epidemiological studies on diet, was the likelihood of measurement error in the diet estimates from the self-administered FFQ, although our FFQ was specially developed and validated in our population.

In summary, this study suggests that high compliance with the MedDiet pattern during pregnancy appears to be favourable for reducing the risk of SGA (< 10th percentile) birth weight, HC and length, suggesting that this diet pattern has a positive effect on foetal growth. These results should be taken into account during the implementation of nutritional intervention programmes, which should focus on a healthy diet like the MedDiet for pregnant women to prevent nutritional deficiencies that might adversely impact the health of the baby.
